# Electrophysiological alterations in motor‐auditory predictive coding in autism spectrum disorder

**DOI:** 10.1002/aur.2087

**Published:** 2019-02-23

**Authors:** Thijs van Laarhoven, Jeroen J. Stekelenburg, Mart L.J.M. Eussen, Jean Vroomen

**Affiliations:** ^1^ Department of Cognitive Neuropsychology Tilburg University 5000 LE Tilburg The Netherlands; ^2^ Department of Child and Adolescent Psychiatry Yulius Mental Health Organization Dordrecht The Netherlands; ^3^ Department of Autism, Yulius Mental Health Organization Dordrecht The Netherlands

**Keywords:** autism spectrum disorder, ERPs, motor‐auditory, predictive coding

## Abstract

The amplitude of the auditory N1 component of the event‐related potential (ERP) is typically attenuated for self‐initiated sounds, compared to sounds with identical acoustic and temporal features that are triggered externally. This effect has been ascribed to internal forward models predicting the sensory consequences of one's own motor actions. The predictive coding account of autistic symptomatology states that individuals with autism spectrum disorder (ASD) have difficulties anticipating upcoming sensory stimulation due to a decreased ability to infer the probabilistic structure of their environment. Without precise internal forward prediction models to rely on, perception in ASD could be less affected by prior expectations and more driven by sensory input. Following this reasoning, one would expect diminished attenuation of the auditory N1 due to self‐initiation in individuals with ASD. Here, we tested this hypothesis by comparing the neural response to self‐ versus externally‐initiated tones between a group of individuals with ASD and a group of age matched neurotypical controls. ERPs evoked by tones initiated via button‐presses were compared with ERPs evoked by the same tones replayed at identical pace. Significant N1 attenuation effects were only found in the TD group. Self‐initiation of the tones did not attenuate the auditory N1 in the ASD group, indicating that they may be unable to anticipate the auditory sensory consequences of their own motor actions. These results show that individuals with ASD have alterations in sensory attenuation of self‐initiated sounds, and support the notion of impaired predictive coding as a core deficit underlying autistic symptomatology. ***Autism Res** 2019, 12: 589–599*. © 2019 The Authors. *Autism Research published by International Society for Autism Research* published by Wiley Periodicals, Inc.

**Lay Summary:**

Many individuals with ASD experience difficulties in processing sensory information (for example, increased sensitivity to sound). Here we show that these difficulties may be related to an inability to anticipate upcoming sensory stimulation. Our findings contribute to a better understanding of the neural mechanisms underlying the different sensory perception experienced by individuals with ASD.

## Introduction

Autism Spectrum Disorder (ASD) is a pervasive neurodevelopmental disorder characterized by deficits in social communication and social interaction and restricted, repetitive patterns of behavior, interests or activities [American Psychiatric Association, 2013; Robertson & Baron‐Cohen, 2017]. ASD has been linked to a range of sensory processing atypicalities, including atypical processing of faces and emotional stimuli [Eussen et al., 2015; Harms, Martin, & Wallace, 2010; Pellicano, Jeffery, Burr, & Rhodes, 2007; Uljarevic & Hamilton, 2013] and hyper‐ and hyposensitivity to perceptual stimuli [Baranek et al., 2013; Robertson & Baron‐Cohen, 2017]. Emerging evidence suggests that many of these atypical sensory experiences reported in ASD may stem from a more general inability to properly integrate sensory information from different sensory sources into accurate and meaningful percepts [Baum, Stevenson, & Wallace, 2015; Beker, Foxe, & Molholm, 2018; Marco, Hinkley, Hill, & Nagarajan, 2011]. Given that sensory cues play a central role in human perception and social interaction, understanding the basis of the atypicalities in sensory processing seen in ASD may very well be a fundamental part of the explanation why individuals with ASD often struggle with social communication and interaction with their environment.

A recently proposed theory that attempts to account for these symptoms, posits that individuals with ASD have impaired predictive coding abilities [Lawson, Rees, & Friston, 2014; Pellicano & Burr, 2012; van Boxtel & Lu, 2013; Van de Cruys et al., 2014]. A key element of the predictive coding theory is the assumption that our brain is constantly generating predictions about the current state of our environment based on previous sensory experience. Collectively, these predictions—or prior expectations, in Bayesian terms—form our internal representation of the world [Friston, 2005; Mumford, 1992]. This internal forward model can be thought of as a probabilistic map that is used to contextualize and inform our perception [Baum et al., 2015; Lawson et al., 2014]. Sensory input is continuously contrasted with our internal predictions. The discrepancy between the sensory input and predictions is reflected in the prediction error [Friston, 2005]. Any unexpected or otherwise informative information is stored in this prediction error, which is then passed up to higher cortical areas, where it is used to readjust and improve the forward model to minimize prediction errors in the future. These predictive mechanisms allow us to anticipate upcoming sensory stimulation and distinguish between expected and unexpected events. The predictive coding account of ASD states that individuals with ASD have a decreased ability to infer the probabilistic structure of their environment [Lawson et al., 2014; Pellicano & Burr, 2012; van Boxtel & Lu, 2013; Van de Cruys et al., 2014]. As a result, they do not possess a precise internal predictive representation of the world around them and may therefore fail to contextualize sensory information in an optimal fashion. Given that statistical learning is vital for acquisition of sensory associations and multisensory integration [Mitchel, Christiansen, & Weiss, 2014; Mitchel & Weiss, 2011; Seitz, Kim, Van Wassenhove, & Shams, 2007], impairments in this process will likely have cascading effects on sensory processing, perception, and social interaction.

One of the most rudimentary predictive coding mechanisms is the ability to distinguish between self‐initiated and external sensory events. This ability is crucial for effective and efficient perceptual organization and interaction with the environment, and has been ascribed to an efference copy/corollary discharge mechanism that enables us to anticipate the sensory consequences of our own motor actions [for review, see Crapse & Sommer, 2008]. A frequently applied approach to examine this predictive mechanism is by recording auditory potentials in a motor‐sensory prediction paradigm. Several studies have shown that the amplitude of the auditory N1 is typically attenuated for self‐initiated sounds, compared to sounds with identical acoustic and temporal features that are triggered externally [Baess, Horváth, Jacobsen, & Schröger, 2011; Baess, Jacobsen, & Schröger, 2008; Bendixen, SanMiguel, & Schröger, 2012; Martikainen, Kaneko, & Hari, 2005]. Within the predictive coding framework, the amplitude of the auditory N1 is assumed to be modulated by the prediction error [Arnal & Giraud, 2012; Friston, 2005]. When an incoming sound matches the prediction, the prediction error is small and thus the amplitude of the auditory N1 is attenuated. For unexpected sounds the prediction error is more pronounced and so the amplitude of the auditory N1 is enlarged. Since self‐initiated sounds are typically experienced as more predictable than externally‐initiated sounds, the prediction error, and hence the N1, for such sounds is typically smaller. From a predictive coding perspective, the N1 attenuation effect for self‐initiated sounds can thus be explained as an attenuation of the prediction error caused by the internal forward model correctly predicting the auditory consequences of one's own motor actions [Martikainen et al., 2005].

If predictive coding is truly impaired in ASD, and individuals with ASD do indeed lack a precise internal forward model to rely on, then perception in ASD is presumably less affected by prior expectations and more driven by sensory input. Following this reasoning, one would expect diminished or absent attenuation of early auditory neural responses by motor‐to‐auditory prediction mechanisms in individuals with ASD. To our knowledge, this has never been formally tested. Hence, the current study examined the neural response to self‐ versus externally‐initiated sounds in individuals with ASD. An experimental paradigm was applied that was similar to those used in previous studies showing robust and consistent motor‐to‐auditory N1 attenuation effects in neurotypical individuals [Baess et al., 2008; Martikainen et al., 2005]. EEG was recorded in a group of older adolescents and young adults with a clinical diagnosis of ASD and in a group of age matched controls with typical development (TD). Motor‐to‐auditory N1 attenuation was examined by comparing event‐related potentials (ERPs) evoked by tones initiated via button‐presses with ERPs evoked by the same tones replayed at an identical pace. Differences between ERPs evoked by self‐ versus externally‐initiated tones were interpreted as top‐down prediction effects [Baess et al., 2011; Baess et al., 2008; Martikainen et al., 2005]. Diminished or absent N1 attenuation, as a neural marker for motor‐sensory predictions, was considered as evidence for impaired predictive coding mechanisms.

## Methods

### *Participants*

Thirty individuals with ASD (8 female, mean age 18.55 years, *SD* = 2.13) and 30 individuals with TD (6 female, mean age 18.83 years, *SD* = 1.32) participated in this study.

Inclusion criteria for participants in both groups were: between 15 and 25 years of age, full scale IQ (FSIQ) > =80, normal or corrected‐to‐normal vision and hearing, absence of physical disabilities and no active use of sedatives 2 days prior to the experiment. Additional inclusion criteria for the ASD group were: a clinical DSM‐IV TR classification of ASD [American Psychiatric Association, 2000] and absence of severe comorbid neurological disorders (e.g., epilepsy). Additional inclusion criteria for the TD group were: absence of any neurological or neuropsychiatric disorder (e.g., ASD, ADHD, epilepsy).

Participants with ASD were recruited at a mental health institution for ASD (de Steiger, Yulius Mental Health, Dordrecht, The Netherlands). At the time of the experiment, all participants in the ASD group were receiving clinical treatment at this mental health institution due to severe mental problems and impaired functioning in activities of daily living linked to ASD. Participants with TD were recruited at Tilburg University and a high school located in the city of Tilburg.

For all participants in the ASD group the clinical DSM‐IV TR classification of ASD was confirmed by two independent clinicians. Additional diagnostic information was retrieved when available, including autism diagnostic observation schedule (ADOS) scores [Lord et al., 2012] and social responsiveness scale (SRS) scores [Constantino & Gruber, 2013]. FSIQ was measured with the Dutch versions of the Wechsler adult intelligence scale (WAIS‐IV‐NL) in participants ≥18 years, and the Wechsler intelligence scale for children (WISC‐III‐NL) in participants <18 years. Demographic details of the ASD group and the TD control group are shown in Table 1. There were no differences in age and gender but the average FSIQ score was higher for the TD group (mean FSIQ 111.97, *SD* = 11.49) compared to the ASD group (mean FSIQ 103.00, *SD* = 16.47), *t*(58) = 2.45, *P* = 0.02.

**Table 1:** Participant Demographics for the Autism Spectrum Disorder (ASD) and Typically Developing (TD) Group

	ASD	TD
Gender^*n.s*.^	22 male, 8 female	24 male, 6 female
Age^*n.s*.^	18.55 (2.13)	18.83 (1.32)
Full scale IQ^*^	103.00 (16.47)	111.97 (11.49)
ADOS	10.11 (5.04) N = 18	‐
SRS	72.91 (9.68) N = 22	‐

n.s. Nonsignificant ^*^ *P* < 0.05 values within parenthesis represent *SD*.

All procedures were undertaken with the understanding and written consent of each participant and—for participants under the age of 18—a parent or another legally authorized representative. Participants with ASD and TD participants that were recruited at the high school were reimbursed with 25 EUR for their participation. TD participants recruited at Tilburg University received course credits as part of a curricular requirement. All experimental procedures were approved by the local medical ethical review board (METC Brabant, protocol ID: NL52250.028.15) and performed in accordance with the ethical standards of the Declaration of Helsinki.

### *Stimuli and Procedure*

Participants were individually tested in a dimly lit and sound attenuated room and were seated in front of a 19‐in. CRT monitor (Iiyama Vision Master Pro 454, Iiyama, Hoofddorp, the Netherlands) positioned at eye‐level at a viewing distance of approximately 70 cm. To ensure that the pace of motor actions was comparable across participants, each participant completed a training session prior to the experiment in which they were trained to adapt their pace to approximately 3000 ms. At the start of the training session, eight 50 ms pure tones of 1000 Hz with an inter stimulus interval of 3000 ms were presented at 70 dB (A) through two loudspeakers located directly to the left and the right of the monitor. Previous motor‐auditory prediction studies typically use headphones for auditory stimulus presentation; however, in the current study loudspeakers were preferred over headphones because they were less obtrusive for the participants in the ASD group. Participants were required to press the left button of a silent mouse with their right index finger in synchrony with the tones, and to continue to press at the same pace after the end of the tone sequence. After 20 button presses (including the eight pacing tones), their mean press interval was presented on the monitor. When the mean interval deviated more than 1500 ms from the required 3000 ms pace, participants were encouraged to speed up or slow down their pace accordingly. The training session was repeated twice for each participant.

**Figure 1:**
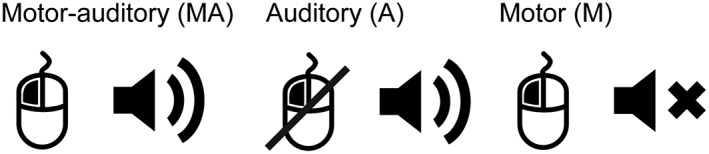
Schematic illustration of the three experimental conditions. In the motor‐auditory (MA) condition, tones were self‐initiated via a button press and the inter‐tap‐interval was recorded. In the auditory (A) condition, the tones were presented at the exact pace of the MA condition and no button presses were allowed. In the motor (M) condition, participants were required to press the button at the same pace as in the MA task, but no tones were presented after each button press.

Three conditions were included in the experiment: motor‐auditory (MA), auditory (A) and motor (M) (Fig. 1). In the MA condition, participants pressed the left mouse button and were encouraged to maintain the previously trained pace of about 3000 ms. After each button press, a 50 ms pure tone of 1000 Hz was presented. Due to hardware restrictions, the temporal delay between the button press and onset of the sound was ~20 ms, which is below the typical detection threshold of motor‐auditory delays [Van Vugt & Tillmann, 2014]. The inter‐press‐interval of the MA condition was recorded to ensure that in the auditory (A) condition, the tones were presented at the exact pace of the MA condition. No button presses were allowed in the A condition and participants were required to refrain from moving their hands, head, fingers or feet in synchrony with the tones. In the motor (M) condition, participants were required to press at the same pace as in the MA condition, but no pure tones were presented after each button press. This condition served as a control condition to rule out the possibility of mere motor activity being a confounder for the expected differences between the A and MA condition [Baess et al., 2008]. Each condition consisted of 120 trials divided across 2 blocks of 60 trials. Block order was quasi‐randomized across participants with the restrictions that an A block was always preceded by an M and MA block, or an MA and M block. Stimulus presentation and button press performance logging was controlled using E‐Prime 1.2 (Psychology Software Tools Inc., Sharpsburg, PA).

To prevent visual EEG activity associated with motor actions, participants were asked to fix their gaze to the monitor and to refrain from looking at the mouse. Participants constantly held their right index finger on the left mouse button and produced mostly isometric muscle contractions without raising their finger before pressing the button to ensure no finger movements were visible in the peripheral visual field. To prevent auditory EEG activity induced by the button presses, we used a mouse specifically designed to produce no clear audible clicks (Nexus SM‐9000). Unlike the switches used in a conventional mouse, the switches used in this mouse lack the typical “click” sound when pressed. In addition, white noise (Hewlett Packard 8057A Precision Noise Generator) was presented during the entire experiment at approximately 60 dB(A) through a single small speaker located at 10 cm behind the mouse, which masked any faint sound originating from the finger movement.

### *EEG Acquisition and Processing*

The EEG was sampled at 512 Hz from 64 locations using active Ag‐AgCl electrodes (BioSemi, Amsterdam, the Netherlands) mounted in an elastic cap and two mastoid electrodes. Electrodes were placed in accordance with the extended International 10–20 system. Two additional electrodes served as reference (Common Mode Sense active electrode) and ground (Driven Right Leg passive electrode). Horizontal electrooculogram (EOG) was recorded using two electrodes placed at the outer canthi of the left and right eye. Vertical EOG was recorded from two electrodes placed above and below the right eye. BrainVision Analyzer 2.0 (Brain Products, Gilching, Germany) and BESA Statistics 2.0 (Brain Electrical Source Analysis, Gräfelfing, Germany) software were used for ERP analyses. EEG was referenced offline to an average of left and right mastoids and band‐pass filtered (0.01–30 Hz, 24 dB/octave). The (residual) 50 Hz interference was removed by a 50 Hz notch filter. Raw data were segmented into epochs of 600 ms, including a 200‐ms pre‐stimulus baseline period. Epochs were time‐locked to the sound onset in the MA and A conditions, and to the corresponding timestamp in the M condition. After EOG correction [Gratton, Coles, & Donchin, 1983], epochs with an amplitude change exceeding ± 150 μV at any EEG channel were rejected and subsequently averaged and baseline corrected for each condition separately. On average 5.35% (*SD* = 7.40) of the trials were rejected. There were no significant differences in rejected trials between groups or conditions (A: TD 4.92, ASD 5.81, MA: TD 3.78, ASD 6.58, M: TD 4.39, ASD 6.61). To facilitate a direct comparison between the A and MA condition, the ERP of the M condition was subtracted from the MA ERP to nullify the contribution of motor activity [Baess et al., 2008; Stekelenburg & Vroomen, 2015].

### *Time Windows and Regions of Interest*

**Figure 2:**
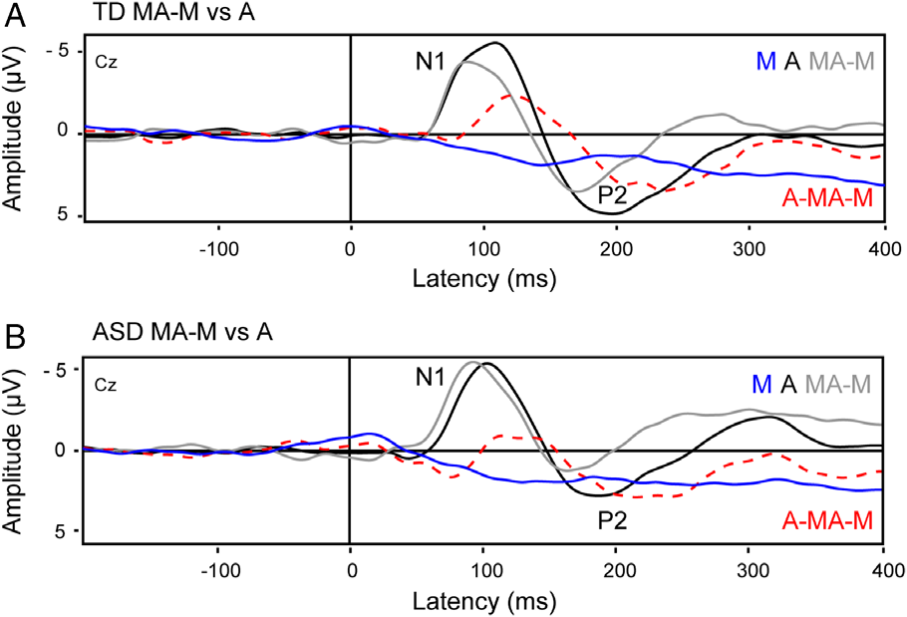
Group‐averaged auditory‐evoked ERPs for the auditory (**A**) and motor‐auditory (MA—M) condition for the TD group (panel **A**) and ASD group (panel **B**). Motor‐auditory ERPs were corrected for motor activity via subtraction of the motor (M) waveform. ERPs were time‐locked to the sound onset in the MA and A conditions, and to the corresponding timestamp in the M condition.

The group‐averaged auditory‐evoked ERPs showed clearly identifiable N1 and P2 responses in the A and MA—M condition in both groups (Fig. 2, panels A and B). Visual inspection of the ERPs showed that only in the TD group, the N1 was attenuated for self‐generated tones in the MA condition compared to the same tones replayed in the A condition. The ERPs from both the ASD and TD group showed that the P2 in the MA condition was attenuated and speeded up compared to the A condition.

To test these observations more formally, a cluster‐based nonparametric permutation procedure was performed to identify time windows and regions of interest for the N1 and P2 [Maris & Oostenveld, 2007]. Difference waveforms reflecting motor‐to‐auditory prediction effects were computed for each group by subtracting MA—M ERPs from A ERPs (i.e., A—MA—M). The time‐course of the difference waveforms of the two groups was compared in the latency range from −200 to 400 ms with a preliminary point‐wise independent samples t‐test identifying clusters that included data points that fell below the cluster alpha level (*P* < 0.05). For each identified cluster, a cluster value was calculated by taking the sum of all the *t*‐values of all data points within that cluster. This preliminary clustering procedure was followed by a permutation procedure that randomly interchanged the cluster values 1000 times. For each permutation, new clusters were identified and the according cluster values were derived. Finally, a new distribution of cluster values was established across all permutations. Clusters were considered significant if the probability of observing a larger cluster value in the new distribution was below the significance level of 0.05.

Clusters revealing significant between group differences in motor‐to‐auditory prediction effects were further explored by comparing ERPs for each condition (A, MA—M) within each group using cluster based permutation tests with parameters similar to those used to examine the A—MA—M difference waveforms. Regions of interest were defined based on the scalp topographies of the time windows identified by the permutation procedures.

## Results

### *Behavioral Performance*

The average button press interval in the MA and M conditions was 2987.30 ms (*SD* = 688.34 ms) and 3133.25 ms (*SD* = 679.35 ms), respectively. Average press intervals for each group and condition were submitted to a repeated measure MANOVA with the within‐subjects variable Condition (MA, A) and between‐subjects factor Group (ASD, TD). The MANOVA produced a significant Condition × Group interaction *F*(1, 58) = 6.51, *P* = 0.01, *η*_p_^2^ = 0.10. Simple main effects tests revealed that for the ASD group, the average press interval was slightly faster (~265 ms) in the MA condition compared to the M condition *F*(1, 29) = 16.15, *P* < 0.001, *η*_p_^2^ = 0.22. However, the average press interval during all conditions was within the required range of 2500–3500 ms, indicating that participants were able to maintain the required pressing pace throughout the entire experiment.

### *Between Group Differences in Motor‐to‐Auditory Prediction (A—MA—M)*

**Figure 3:**
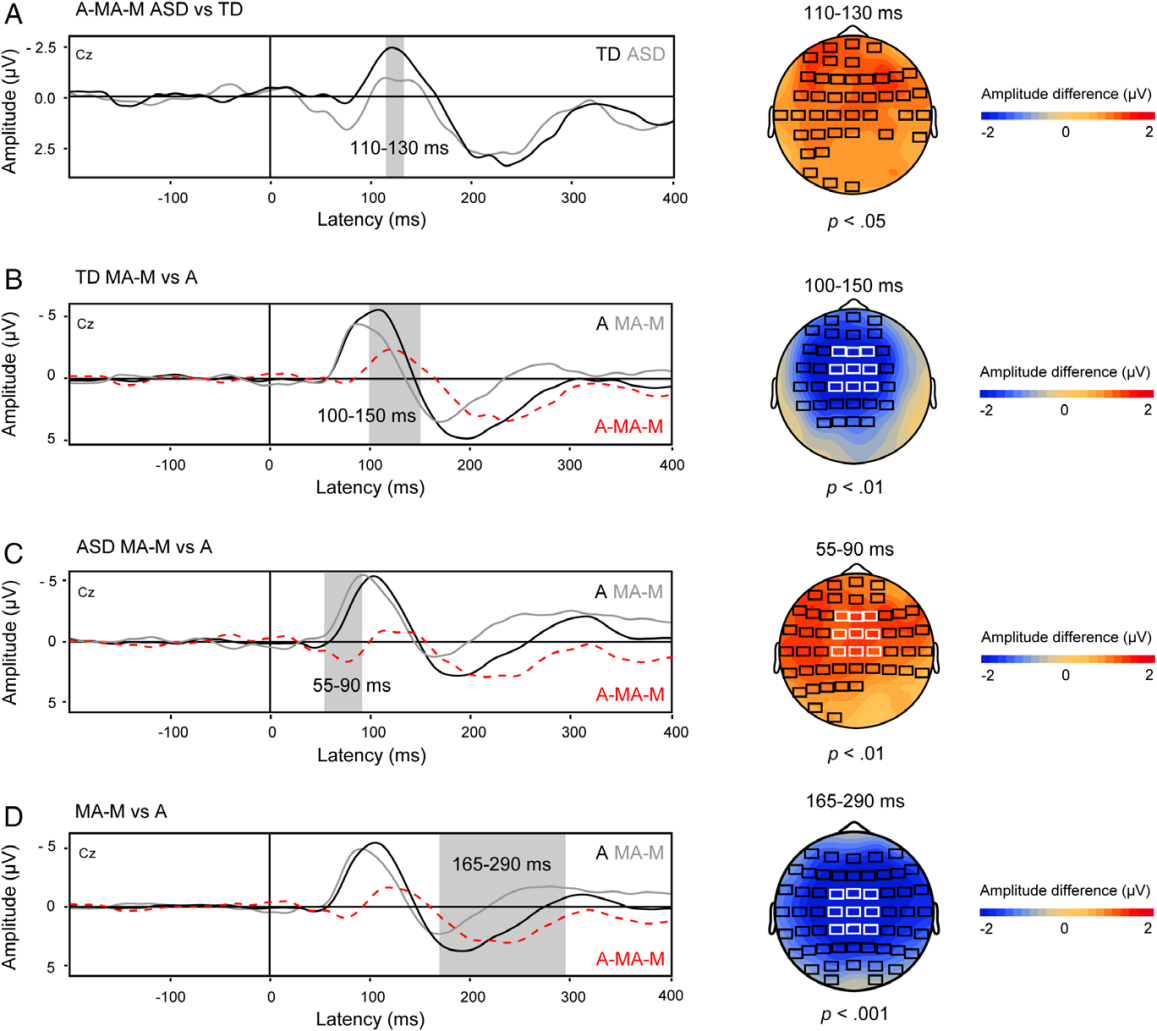
Results of the cluster‐based permutation tests. Panel **A**: Group‐averaged difference waveforms reflecting motor‐to‐auditory prediction effects were computed for each group by subtracting MA—M ERPs from A ERPs (i.e., A—MA—M). Waveforms were time‐locked to the sound onset in the A and MA conditions, and to the corresponding timestamp in the M condition. A time window of interest was identified in the latency range from 110 to 130 ms showing a significant difference (*P* = 0.03) between the ASD and TD group that was most pronounced over fronto‐central electrodes. The between‐group difference in the 110 and 130 ms time window was further explored by comparing ERPs for each condition within each group (panels **B** and **C**). Panel **B**: For the TD group, a time window of interest was identified in the latency range from 100 to 150 ms indicating a significant difference between the A and MA—M condition that was most pronounced over fronto‐central electrodes. Panel **C**: For the ASD group, an earlier time window of interest was identified in the latency range from 55 to 90 ms indicating a significant difference between conditions that was most pronounced over fronto‐central electrodes. Panel **D**: Waveforms reflecting overall neural activity across groups were computed for each condition to examine differences in P2 mean activity between the A and MA—M condition. A time window of interest in the latency range from 165 to 290 ms was revealed showing a significant difference between the A and MA—M condition that was most pronounced over central electrodes. *Scalp topographies*: Black rectangles indicate electrodes showing a significant difference in motor‐to‐auditory prediction effects (panel **A**) or a significant difference in mean activity between the A and MA—M condition (panels **B**, **C**, and **D**). White rectangles depict electrodes included in confirmatory parametric analysis.

The cluster‐based permutation test revealed a time window of interest for the N1 in the latency range from 110 to 130 ms showing a significant difference (*P* = 0.03) between the ASD and TD group that was most pronounced over fronto‐central electrodes (Fig. 3, panel A). No other time windows of interest were identified, indicating that the difference in mean activity between self‐ versus externally‐initiated tones in the P2 latency range was similar for both groups.

### *N1 Responses to Self‐ versus Externally‐Initiated Tones*

#### *N1 time window*

To further explore the between‐group difference in the 110–130 ms time window of interest for the N1, ERPs for each Condition (A, MA—M) were compared within each group using cluster based permutation tests similar to those used to examine the A—MA—M difference waveforms. For the TD group, the permutation tests revealed a significant difference between the A and MA—M condition in the latency range from 100 to 150 ms (Fig. 3, panel B). Mean activity in this time window was significantly attenuated for self‐initiated compared to externally‐initiated tones (*P* < 0.01). Importantly, this time window showed substantial overlap with the previously identified 110–130 ms time window of interest. For the ASD group, there was no significant difference between conditions in the 110–130 ms time window. However, an earlier time window of interest was identified (Fig. 3, panel C). Mean activity in the latency range from 55 to 90 ms was significantly *increased* (i.e., more negative) for self‐initiated compared to externally‐initiated tones (*P* < 0.01). Given the morphology of the ERPs, this increase in N1 mean activity likely reflects a difference in onset and latency.

To further examine the observed amplitude and latency effects, additional confirmatory parametric testing was carried out on the peak amplitude and peak latency values in the latency range from 55 to 150 ms. This latency range was selected to include the previously identified time windows of interest for each group (i.e., ASD: 55–90 ms, TD: 100–150 ms). Based on the scalp topographies of the time windows identified by the permutation procedure (Fig. 3, panel B and C), a fronto‐central region of interest (ROI) including nine electrodes with FCz at its center was defined. Individual N1 peak amplitude and peak latency values within the 55–150 ms time window were calculated for each condition and electrode and submitted to repeated measures MANOVAs with the within‐subjects variables Condition (A, MA—M) and Electrode (Cz, C1, C2, FCz, FC1, FC2, Fz, F1, F2) and between‐subjects factor Group (ASD, TD).

#### *N1 amplitude*

**Figure 4:**
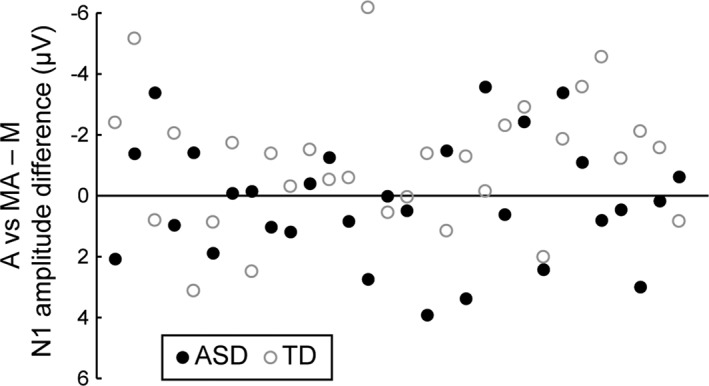
Scatter plot showing individual differences in N1 amplitude between the A and MA—M condition in the fronto‐central ROI (Cz, C1, C2, FCz, FC1, FC2, Fz, F1, F2). Negative values indicate N1 attenuation.

The MANOVA for N1 amplitude produced a significant Condition × Group interaction, *F*(1, 58) = 5.70, *P* = 0.02, *η*_p_^2^ = 0.09 and a main effect of Electrode, *F*(8, 51) = 18.32, *P* < 0.001, *η*_p_^2^ = 0.74. The main effect of Electrode was further examined with post hoc paired samples *t*‐tests (Bonferroni corrected), which showed that N1 amplitude was less negative at C1, Cz, and C2 than at FC1, FCz, FC2, Fz, and F2 (all *P* values < 0.05), and less negative at F1 than at FCz, Fz, and F2 (all *P* values < 0.05). The Condition × Group interaction was further explored with simple main effects tests examining the effect of Condition within each Group. For the TD group, there was a main effect of Condition, *F*(1, 29) = 8.06, *p* < 0.01, *η*_p_^2^ = 0.12, indicating that the amplitude of the auditory N1 was significantly attenuated for self‐initiated tones in the MA—M condition compared to the same tones replayed in the A condition. There was no main effect of Condition for the ASD group, *F*(1, 29) = 0.29, *P* = 0.59, *η*_p_^2^ = 0.005, indicating that self‐initiation of the sound did not modulate the amplitude of the auditory N1 (see Fig. 4 for individual N1 amplitude differences between the A and MA—M condition).

To ensure that the difference in FSIQ between the ASD and TD group was not a confounding factor for the absent N1 attenuation in the ASD group, a post hoc partial correlation analysis controlling for group membership was conducted correlating individual N1 amplitude difference between the A and MA—M condition in the fronto‐central ROI to FSIQ. This analysis revealed that the extent of N1 attenuation was not affected by FSIQ (r = 0.16, *P* = 0.22), thereby ruling out FSIQ as a confounding factor for the absent N1 attenuation in the ASD group.

#### *N1 latency*

The MANOVA for N1 latency showed a main effect of Condition, *F*(1, 58) = 30.21, *P* < 0.001, *η*_p_^2^ = 0.34. The N1 was speeded up by ~9 ms in the MA condition compared to the A condition (A: 105.18 ms MA—M: 96.58 ms). There was no main effect of Group or Condition × Group interaction, indicating that the N1 for self‐initiated tones was speeded up similarly in the ASD and TD group. This confirmed that the difference in mean activity between the A and MA—M condition in the 55–90 ms time window for the ASD group (as revealed by the cluster based permutation tests) was indeed due to a temporal shift of the rising flank of the N1—and not due to a difference in N1 amplitude between modalities.

### *P2 Responses to Self‐ versus Externally‐Initiated Tones*

#### *P2 time window*

The initial permutation test on the A—MA—M difference waveforms revealed no significant differences between the ASD and TD group in the P2 latency range. Visual inspection of the ERPs (Fig. 2, panels A and B) suggests that in both groups, the mean activity in the P2 latency range was less positive and speeded up in the MA—M condition compared to the A condition. To verify this observation, neural auditory activity across both groups was computed for each condition, and submitted to a cluster‐based permutation test. This procedure revealed a time window of interest in the latency range from 165 to 290 ms showing a significant difference (*P* < 0.001) between the A and MA—M condition that was most pronounced over central electrodes (Fig. 3, panel D). Confirmatory parametric testing was carried out on the peak amplitude and peak latency values in this time window in a central ROI including Cz and eight directly surrounding electrodes. Individual P2 peak amplitude and peak latency values within the 165–290 ms time window were calculated for each condition and electrode and submitted to repeated measures MANOVAs with the within‐subjects variables Condition (A, MA—M) and Electrode (CPz, CP1, CP2, Cz, C1, C2, FCz, FC1, FC2).

#### *P2 amplitude*

The MANOVA for P2 amplitude produced a significant Condition x Electrode interaction, *F*(8, 52) = 2.68, *P* = 0.02, *η*_p_^2^ = 0.29. This interaction was further explored with simple main effects tests examining the effect of Condition at each Electrode. In all electrodes, P2 amplitude was significantly attenuated in the MA condition compared to the A condition (all *P‐*values < 0.03, average amplitude difference 1.53 μV).

#### *P2 latency*

The MANOVA for P2 latency showed a main effect of Condition, *F*(1, 59) = 46.41, *P* < 0.001, *η*_p_^2^ = 0.44, indicating that the P2 was speeded up by ~18 ms in the MA condition compared to the A condition (A: 192.43 ms MA—M: 173.99 ms).

### *Summary*

N1 latency and attenuation effects for self‐initiated tones were found in the TD group. In the ASD group, the auditory N1 for self‐initiated tones was speeded up but—crucially—not attenuated, whereas the P2 for self‐initiated tones was speeded up and attenuated in both groups.

## Discussion

The current study tested the predictive coding account for autistic symptomatology by comparing the neural response to self‐ versus externally‐initiated tones in individuals with ASD and TD. The data revealed clear group differences in the neural correlates of internal motor‐to‐auditory prediction mechanisms. Significant N1 attenuation effects were found in the TD group, indicating that a forward model predicted the auditory consequences of their motor actions. These results are consistent with the literature on typical electrophysiological indicators for predictive processing in audition [Baess et al., 2008; Bendixen et al., 2012]. Most importantly, self‐initiation of the tones did not attenuate the auditory N1 in the ASD group. The extent of N1 attenuation is presumed to be positively correlated with the accuracy of the prediction of the upcoming stimulus [Arnal & Giraud, 2012; Friston, 2005]. The absence of N1 attenuation in the ASD group could thus indicate that, even in a relatively stable context, individuals with ASD experience difficulties in anticipating upcoming sensory events and seemingly process every stimulus afresh—rather than mediated by prior expectation. The current results could be indicative of impaired motor‐to‐auditory predictions in ASD, and support the impaired predictive coding account of autistic symptomatology [Lawson et al., 2014; Pellicano et al., 2007; van Boxtel & Lu, 2013; Van de Cruys et al., 2014].

Although the N1 was not attenuated for self‐initiated tones in the ASD group, it was speeded up similar as in the TD group. Previous studies have shown that N1 latency facilitation only occurs if the preceding stimulus provides reliable predictive information about the identity of the upcoming sound [Arnal, Morillon, Kell, & Giraud, 2009; Paris, Kim, & Davis, 2017]. The similar N1 latency facilitation in both the ASD and TD group may thus suggest that predictions regarding the identity of the tones were intact in the ASD group. Yet the absence of N1 attenuation in the ASD group indicates that auditory predictions for self‐initiated tones were not enhanced by the cues provided by the preceding motor action. It could be speculated that participants in the ASD group failed to infer the temporal relationship of the tones relative to the button presses. As a result, predictions about the onset of self‐initiated tones may have been impaired. This interpretation aligns with recent observations of impaired multisensory temporal acuity in ASD [Noel, De Niear, Stevenson, Alais, & Wallace, 2017; Stevenson et al., 2016]. It should be noted, however, that in TD individuals, significant (albeit smaller) auditory N1 attenuation effects have been reported for self‐initiated sounds with unpredictable timing and content [Baess et al., 2008; Knolle, Schröger, & Kotz, 2013b]. Others have shown that tones triggered by a key‐press elicit a smaller N1 than tones following a visual cue with predictable timing [Lange, 2011], suggesting that the attenuated N1 to self‐initiated tones is not merely caused by the fact that self‐initiation provides a highly reliably cue for tone onset. Thus, N1 attenuation for self‐initiated sounds may in part reflect a more general predictive mechanism [Baess, Widmann, Roye, Schröger, & Jacobsen, 2009; Martikainen et al., 2005; Sanmiguel, Todd, & Schröger, 2013]. Based on the current study it cannot be resolved whether the absence of N1 attenuation to self‐initiated sounds in the ASD group was caused by impairments in temporal‐, identity‐, or general prediction. In a future study it would therefore be interesting to investigate the relative contribution of temporal‐ and identity predictions in individuals with ASD by contrasting a single sound condition with a random sound condition [cf., Baess et al., 2008].

For both the TD and ASD group, the N1 for self‐initiated sounds was followed by an attenuated and speeded up P2 response. Although N1 attention effects are often accompanied by a suppression of the P2 component, the P2 can be functionally dissociated from the N1 [Crowley & Colrain, 2004]. While the exact functional interpretation of the auditory P2 component is still unclear, it has been argued that an attenuated P2 response to self‐ initiated tones may reflect the conscious post hoc realization that a sound closely following a button press must have been self‐initiated—as opposed to an attenuated N1 response, which reflects the effect of an automatic prospective internal forward prediction mechanism [Knolle, Schröger, & Kotz, 2013a]. The current data could therefore indicate that, even though individuals with ASD are aware of the fact that auditory stimulation can be self‐initiated, they are unable to effectively use the predictive information provided by their own motor actions to anticipate the auditory sensory consequences of those actions.

Previous studies have shown that increasing attention toward an auditory stimulus may result in higher N1 amplitudes [Lange, Rösler, & Röder, 2003], whereas drawing attention away may attenuate the N1 response [Horváth & Winkler, 2010]. It could therefore be argued that increased attention to self‐initiated sounds—relative to externally‐initiated tones—may have resulted in an amplitude increase of the auditory N1 in the ASD group. An argument against this view is that attenuation of the P2 was similar in the ASD and TD group, indicating that a potential difference in allocation of attention between self‐ and externally‐initiated tones was likely similar in both groups. Still, the N1 was significantly attenuated—rather than enlarged—in the TD group, thereby rendering sustained attentional differences between experimental conditions an unlikely account for the absence of N1 attenuation in the ASD group. Furthermore, this attentional account was specifically tested and refuted in a study using a N1 suppression paradigm, where self‐ and externally‐initiated sounds were randomly intermixed and presented within the same block [Baess et al., 2011]. Because externally‐initiated sounds occurred at unpredictable intervals within the same block as self‐initiated sounds, ERP differences between self‐ and externally‐initiated sounds could not stem from a difference in task demands between the experimental conditions. The results showed an even larger N1 attenuation effect for self‐initiated sounds than typically observed in a blocked N1 suppression paradigm (as used in the current experiment), indicating that N1 attenuation for self‐ versus externally‐initiated sounds is independent of attention. It can also be argued that the difference in N1 attenuation between the ASD and TD group was due to a difference in allocation of attention between modalities during self‐initiation of the tones. Increased attention to the auditory tones—relative to the motor act—may have led to an amplitude increase of the auditory N1 in the ASD group. However, this attentional account was also examined and refuted in a recent study [Timm, SanMiguel, Saupe, & Schröger, 2013]. Using a similar mixed N1 suppression paradigm as Baess et al. [2011], allocation of attention was manipulated block‐wise to either the sound, the motor act or to a visual stimulus. The results showed similar N1 attenuation effects for self‐initiated sounds in all three attention conditions.

Taken together, these findings imply that the lack of N1 attenuation for self‐initiated tones in the ASD group cannot be explained by potential differences in allocation of attention, but instead, more likely reflects the activity of an impaired motor‐to‐auditory prediction mechanism.

### *Future Directions*

If individuals with ASD are indeed unable to anticipate the sensory consequences of their own actions, this raises the question if their ability to predict actions of other individuals is impaired as well. Given that other people's behavior is arguably more difficult to predict than self‐initiated actions, and the fact that individuals with ASD have great difficulty with understanding the thoughts and emotions of their own and those of others [Robertson & Baron‐Cohen, 2017], it is reasonable to assume that this might indeed be the case. There is indeed evidence suggesting that individuals with ASD have specific deficits in attributing mental states to others (i.e., mentalizing), whereas processing of lower‐level social information is intact [David et al., 2010; Sebanz, Knoblich, Stumpf, & Prinz, 2005; Zwickel, White, Coniston, Senju, & Frith, 2011]. Future studies should address if these findings can be linked to electrophysiological alterations. Previous studies have reported that in TD individuals, attenuation effects of auditory potentials are not limited to the motor‐auditory domain but are found in other inter‐sensory domains as well. For example, seeing someone performing a handclap provides predictive information about the upcoming sound. Several studies have demonstrated that such anticipatory information attenuates and speeds up the auditory N1 and P2 [Stekelenburg & Vroomen, 2007, 2012; Vroomen & Stekelenburg, 2010]. Others have reported that a rare omission of a sound that is predictable by anticipatory visual information typically induces an early negative response in the EEG during the period of silence where the sound was expected [Stekelenburg & Vroomen, 2015; van Laarhoven, Stekelenburg, & Vroomen, 2017]. In a future study, it would therefore be interesting to investigate if the alterations in motor‐to‐auditory prediction observed in the current group of individuals with ASD extend to the visual–auditory domain.

One particular brain region of potential interest for future work on motor‐to‐auditory prediction in ASD is the cerebellum. Findings from two recent studies examining N1 attenuation to self‐initiated tones in patients with lesions in the cerebellum suggest that this particular brain region is involved in the generation of motor‐to‐auditory predictions [Knolle, Schröger, Baess, & Kotz, 2012; Knolle et al., 2013a]. Using a paradigm similar to that of the current study, it was found that the N1 to self‐initiated tones was attenuated in controls but not in patients with cerebellar lesions, while P2 attenuation due to self‐initiation was similar in both groups. Although the clinical phenomenology of the populations included in these studies and the current study is fundamentally different, the similarities in ERPs between the cerebellar lesion patients and the current sample of individuals with ASD are noteworthy. While there is in fact an emerging literature on cerebellar alterations in ASD (for review, see Hampson & Blatt, 2015], future neuroimaging studies should examine if these similarities in neural correlates of motor‐to‐auditory prediction mechanisms indeed stem from deficits in the same underlying neural networks.

## Conclusions

The current results confirm our hypothesis that individuals with ASD show alterations in sensory attenuation of self‐initiated sounds. Specifically, predictive cues provided by button presses did not attenuate the auditory N1 in our sample of individuals with ASD. The current data indicate that motor‐to‐auditory prediction may be impaired in ASD, and support the notion of impaired predictive coding as a core deficit underlying atypical sensory processing in ASD.
